# Editorial: Insights in healthcare professions education: 2021

**DOI:** 10.3389/fmed.2022.1054572

**Published:** 2022-11-15

**Authors:** Lynn V. Monrouxe, Jacqueline G. Bloomfield

**Affiliations:** Faculty of Medicine and Health, The University of Sydney, Camperdown, NSW, Australia

**Keywords:** interprofessionalism education, social media use, knowledge synthesis, assessment, tolerance of uncertainty, transitions, imposter syndrome

The Healthcare Professions Education specialty section of Frontiers in Medicine opened for submissions in May 2021, with this interesting collection of articles being the first to be developed. As we discussed in our previous editorial, healthcare professions education is a widely diverse field covering multiple topics (Monrouxe and Bloomfield). This research collection is no exception. Thus, we cover a range of considerations within the field of healthcare professions education broadly grouped into the themes of interprofessionalism, social media use, knowledge synthesis, assessment and theory-driven research. Furthermore, our publications span a range of different contributions including opinion articles, original research and reviews.

## Interprofessionalism

Interprofessionalism remains a hot topic in healthcare professions education, being of central importance to successful teamworking and collaboration across a diverse range of healthcare settings. Furthermore, we see diversity in terms of the articles we have grouped into this theme as a few cross-cut our other themes (with some reporting incidental findings around interprofessionalism). This attests to the complexity and interdisciplinarity of healthcare education.

Samuriwo comments on how the success of interprofessional collaboration in healthcare practice is to some extent dependent upon interprofessional education during the undergraduate years. He points out that these interprofessional concepts can be thought of as “*muddy zones of practice*” (1): complex and uncertain areas that, for successful outcomes, requires us to think through different lenses (e.g., beliefs, sociocultural influences) due to the diversity inherent across different professional groups' practices (Monrouxe and Bloomfield). Indeed, interprofessional learning appears to be one of the numerous success factors of the undergraduate nursing interactive curriculum, as reported by Fooladi et al. thinking through different lenses and addressing students' stereotypes, developing their communication skills. We also see interdisciplinary collaboration being fostered through theory-driven approaches in Mukhalalati et al.'s review of theory-use in healthcare professionals' programme development. Thus, simulation exercises using the constructs of self-efficacy is found to enhance problem-solving, communication skills and clinical competency (2). Additionally, educators drew on Communities of Practice theory to create interprofessional teamworking during clinical placements (3). Umar et al.'s review of healthcare students' pandemic-related volunteering also reveals that a key benefit from undertaking these activities includes interprofessional collaboration. Furthermore, in their review of virtual simulation learning over a 2-year period (2020–2021), Wu et al. report two studies of interprofessional training across a range of healthcare students (medicine, nursing, pharmacy, occupational therapy, physiotherapy, medical social work). Together, these two studies suggest that immersive technologies are useful for delivering high-quality interprofessional training. Perhaps, therefore, the use of virtual reality avatars, giving healthcare students a chance to inhabit the body of others, is one way of clarifying this *muddy zone* of interprofessional practice highlighted by Samuriwo.

## Social media use

The use of social media by healthcare students is the focus of two of our articles. Bansal highlights that, for the past 10 years, the exponential growth in Open Access online educational materials for preclinical and clinical medical students has resulted in over 90% of students using these resources regularly (10). Furthermore, this global shift in approaches to learning draws on a diverse range of resources including social media platforms (e.g., podcasts, Twitter, YouTube) bringing with it some challenges alongside the obvious benefits associated with flexible learning. Challenges around the educational quality of the resources include developers' skills, understanding of instructional design and sustainability. Future submissions focusing on research addressing such challenges are welcome, alongside other commentaries on the inclusion of high-quality Open Access online educational materials for healthcare professional students.

While Bansal focuses on the educational benefits and challenges of social media platforms, Guraya et al. consider the issue of students' e-professionalism lapses which: “merely addresses professionalism in the online world” ((4), p. 170). The issue of e-professionalism lapses by healthcare students is of international concern with students openly violating professional standards involving a diverse range of stakeholders including breaches of patient confidentiality, being derogatory about patients, seniors and peers, and even “partying and drinking alcohol in inappropriate attires” (Guraya et al., p. 2). Utilizing the Theory of Planned Behavior as an underlying construct (5), Guraya et al.'s mixed-methods evaluation of an intervention designed to increase awareness and behaviors shows promising results with participants' displaying (among other things) strong *intentions* to be digitally professional (e.g., raising concerns, taking responsibility seriously and being digitally reflective). The issue of e-professionalism is of great importance in healthcare professional education, and there is real gap in our understanding of the deeper underlying issues around students' online lapses. We encourage researchers to address this gap in future submissions to our section.

## Knowledge synthesis

Partly as a response to the call for evidence based education, knowledge synthesis approaches across healthcare professions research have increased 10 times more than other research approaches over the past 20 years or so (6). Echoing this trend, we have four articles in our collection, two scoping and two systematic reviews, each providing us with an overview of the state of research on specific educational interventions: virtual simulation (Wu et al.), the medical humanities (Hoang et al.), the application of learning theory in healthcare professions education (Mukhalalati et al.), and students' extra-curricular responses to the pandemic (Umar et al.).

Thus, Wu et al. reveal that, in a brief 2-year period, 92 articles focusing on virtual simulation in medical education settings have been published from 25 countries: although predominately Europe (45%) and North America (33%). The largest category of work reports on undergraduate students' surgical training (38%) covering areas such as endoscopic, orthopedics/bone and neurosurgery/neuroanatomy. Other areas serviced by virtual simulation interventions include emergency and pediatric emergency medicine training, basic medical sciences, medical radiation/imaging, puncture/catheterization and (as highlighted earlier) interprofessional healthcare. Across these diverse virtual simulation use cases, over half use virtual reality deploying headsets or goggles and/or hand controllers, and with the vast majority demonstrating evidence of an increase in learners' knowledge or skills (Wu et al.). Where evidence is lacking is in the wider “so what” for change in practice and patient benefit.

The medical humanities comprise a diversity of humanity-based interventions aimed at counterbalancing the relatively “sterile” and clinical focus of healthcare training (an intrinsic rationale), developing communication and empathy skills (an instrumental rationale), bringing an analytical/questioning perspective health practices (a critical rationale), and/or making the relevance of the humanities disciplines and enquiry obvious to healthcare professions education and practice (an epistemological rationale) (7). Hoang et al. reviewed the state of research around the medical humanities across a 20-year period in one Asian country, Taiwan, identifying 17 articles. The majority of articles from their review drew on the instrumental and intrinsic rationales, reporting on the efficacy of a range of interventions including visual arts, narrative, reflective practice and fieldwork. However, evidence for efficacy appears to be mixed. Eight studies reported positive increases for attitudes around treating patients with greater humanity or being more creative and critical in their studies, or increases in knowledge and skills in critical thinking, reflective writing and empathy. Despite this, three studies failed to deliver the expected outcomes of greater empathy or ethical decision-making. As with Wu et al. there is a lack of research examining the higher levels of behavioral change and impact on patient benefit. We encourage researchers to consider these higher levels of impact when designing future studies and welcome submissions reporting on the degree to which interventions reach these outcomes.

As Lewin's Maxim states, in the case of applied research, “*there is nothing as practical as a good theory*” ((8), p. 118). Mukhalalati et al. sought to identify how social learning theories are utilized in healthcare professions education programmes (2011–2020). They found only nine studies reporting this use, drawing on Bandura's Social Learning Theory and Lave and Wenger's Communities of Practice. Studies are predominately in nursing programmes but also medicine, pharmacy and multi-disciplinary contexts, focusing on teaching and learning, assessment and curriculum design. This review highlights the relative absence of explicit work around theory-use in programme development. Indeed, the authors comment on how many articles were excluded through failing to explain *how* theory were used, or because they theories were used as exploratory lenses, looking *onto* research data (rather than using theory to *develop* interventions). Future research using theory to drive curricula development is encouraged and we welcome manuscripts with this in mind.

Umar et al. found 41 studies in a 22 month period, identifying the motivations, benefits, activities and barriers around health professional students' pandemic-related volunteering. Over 75% of these studies focused on medical students. Diverse activities include working in hospitals (e.g., emergency departments and admissions), call centers, contact tracing/testing and online consultations. Key considerations around students' engagement in these activities comprise a range of internal (self) and external (societal/healthcare system) rationales including their sense of moral responsibility, possibilities for learning, adequate PPE provision, appropriate knowledge/skills and financial remuneration: many of which were also cited as benefits arising from participating. Participating in these activities also facilitate psychological wellbeing. Although it is likely, as we move forward into a post-pandemic area, that such volunteering will change, we wonder what the longer-term educational impact of such volunteering is. For example, at an individual level, do students feel better prepared for practice? At an institutional level, will undergraduate healthcare programmes encourage (or develop) post-pandemic activities in this likeness? Or will these practices merely fade away? We are interested in hearing more about the outcomes and evolution of these practices over time.

## Assessment

Assessment in healthcare education is complex and diverse, as evident by the different purposes of assessment, the various ways in which assessment can be conducted, and the different tools that can be used to facilitate assessment (e.g., checklists, marking guides, proformas). Further to this diversity is the variation in assessors' backgrounds and roles. Some are totally university-based, while others share their time between university and clinically-based roles. It is because of this diversity that educators continue to grapple with issues of variability in clinical assessment and subjectivity among assessors. This raises concerns about cognitive bias, as well as having assessment methods that are fit for purpose. These issues have informed both studies in our collection grouped together under the Assessment theme.

Recently, the notion of the *prototype intern* has emerged. Grounded in the heuristic mental construct of assessors' professional and clinical expectations of an *ideal new graduate* working within their team, the concept of a prototype intern influenced Malau-Aduli et al. to conduct an interesting study, emphasizing the diverse complexity of an OSCE assessment. Informed by an interpretivist paradigm, 20 assessors from ten medical schools across Australia and New Zealand engaged in focus group discussions exploring how the concept of the prototype intern influenced their judgements during senior medical students' exit-level OSCEs. Findings suggest a complex but interrelated influence of academic and workplace constructs on assessors' judgements. Thus, individual assessors balanced the task-orientated expectations of the academic system (i.e., outcomes of knowledge, skills and behaviors), with professional expectations of the clinical workplace (e.g., respect, reliability, teamwork and trustworthiness). Malau-Aduli et al. also identified variation among individual assessors. Those who are more clinically experienced relied on their mental construct of the prototype intern than those who are less experienced. In concluding, the researchers suggest the need for explicit assessor training aimed at reducing unconscious bias and identify the need for further research in this area. We look forward to future submissions that address this gap.

We also see the diversity of assessment methods in health profession education in the longitudinal study reported by Liu et al. Again, focusing on medical education, these researchers analyzed retrospective educational data collected over a four-year period from multiformat in-training examinations (ITE) of emergency medicine residents at a large teaching hospital in Taiwan. With the goal of determining the validity, reliability, cost and learner satisfaction of the multiformat ITE, Liu et al. found that that the incorporation of different assessment formats in the ITE [written tests, oral examinations and high-fidelity simulations (HFS)] demonstrated good reliability. They also found that the inclusion of HFS as part of the ITE expanded the domains that could be tested, in that they added a clinically authentic assessment. The researchers' identified, however, the high potentially inhibitive costs associated with HFS and the need for educators to understand the strengths and weaknesses of each type of assessment, and the context in which they are to be used. This is an issue that all healthcare educators involved in the development of assessment strategies should consider.

## Theory-driven

The final three articles in our first collection align well with the diverse nature of research in the field of health professional education in that they address a broad and fascinating array of topics that include: imposter syndrome and its relationship to self-esteem in medical students (Naser et al.); the impact of gendered transitions on female surgeons (Offiah et al.); and factors influencing the tolerance by medical students of uncertainty (Stephens et al.). Not only are these topics varied, although, commonly linked in that each is clearly underpinned by a specific theory, drawing on participants' personal experiences, but all have been conducted using distinctively different study designs: a cross-sectional questionnaire (Naser et al.); narrative inquiry (Offiah et al.); and a qualitative longitudinal design that involved analysis of reflective diaries and semi-structured interviews (Stephens et al.).

In a study of 290 medical students from 28 different nationalities studying at an Irish medical college situated in Bahrain, Naser et al. sought to assess the prevalence of imposter syndrome and its relationship to self-esteem. Findings indicated that imposter syndrome was higher among students with low self-esteem and that students from Gulf corporation countries (Bahrain, Kuwait, Oman, Saudi Arabia and the United Arab Emirates) had the highest levels of self- esteem. Interestingly, the researchers found no differences between gender. This finding is dissimilar to other studies which have shown an association between imposter phenomenon and female medical students (9, 10).

Gender was also the focus of a fascinating study that explored transition across surgical training as experienced by female surgeons within Scotland and Ireland (Offiah et al.). Drawing on Multiple and Multidimensional Transitions theory (MMT), Offiah et al. using qualitative methods, found that female surgeons experienced four intersecting transitions within their surgical training trajectory, with gender having a considerable impact. These included maternity leave transitions, part-time training/working transitions, leadership transitions and transitions out of surgery. Each bringing with it a multitude of challenges. In discussing these findings, the researchers highlight the need for greater awareness around these factors and for educators, leaders and policy-makers to develop interventions and services to aimed at addressing the needs of female surgeons as they progress through their careers.

In the third of these articles, the construct of uncertainty tolerance was the focus of an Australian qualitative, longitudinal study of medical students (Stephens et al.). According to Hillen et al. (11), after perceiving uncertainty, a person will appraise and respond to it across cognitive, emotional and behavioral domains with different moderators influencing either their perception of the uncertainty or their response to it. Uncertainty tolerance is now considered an essential trait for medical graduates, with research demonstrating a link between lower uncertainty tolerance in physicians and medical students and negative outcomes such as paternalistic attitudes toward patients, increased use of healthcare resources and a higher risk of burnout. Stephens et al. used semi-structured individual and group interviews and analyses of diary entries to explore the factors perceived by medical students as moderating their perceptions and responses to uncertainty in the clinical context. A broad and diverse range of moderators, which were categorized as individual factors, sociocultural factors and academic factors were identified as having either a positive or negative impact on the medical students' perceptions of uncertainty. A fourth moderator, reflective learning, was reported to be a positive influence. In discussing the implications of these findings for educators, the researchers emphasize the need to incorporate opportunities for reflection within teaching and learning as well as the importance of educators helping students' navigation of uncertainty. Further research is now needed, and we welcome future papers that address this key topic of uncertainty tolerance.

## Conclusion

In our roles as Chief Specialty Editors for the Healthcare Professions Education section of Frontiers in Medicine we have been delighted at the diversity of high-quality submissions that have been received over our first year. We take this opportunity to sincerely thank the reviewers and handling editors for their work, without which would make the success of the section impossible. We look forward to a diversity of future publications that continue to challenge, to educate and to advance knowledge in this important field.

## Author contributions

LM lead the writing of the manuscript, theming the articles in the section, structuring the paper, and finalized the manuscript to completion. LM and JB summarized the articles. Both authors approved the final manuscript for submission.

## Conflict of interest

The authors declare that the research was conducted in the absence of any commercial or financial relationships that could be construed as a potential conflict of interest.

## Publisher's note

All claims expressed in this article are solely those of the authors and do not necessarily represent those of their affiliated organizations, or those of the publisher, the editors and the reviewers. Any product that may be evaluated in this article, or claim that may be made by its manufacturer, is not guaranteed or endorsed by the publisher.
